# Covalent organic frameworks-based materials for antibiotics fluorescence detection

**DOI:** 10.1016/j.heliyon.2024.e33118

**Published:** 2024-06-17

**Authors:** Mingyang Ji, Jiani Li, Anan Liu, Dongge Ma

**Affiliations:** a Department of Chemistry, School of Light Industry Science and Engineering, Beijing Technology and Business University, 100048, Beijing, China; b Basic Experimental Centre for Natural Science, University of Science and Technology Beijing, Xueyuan Road 30, Beijing, 100083, China

## Abstract

Antibiotics play a vital role in safeguarding people's health since most bacterial infection can be efficiently controlled and cured by treating with suitable antibiotics. However, excessive use of antibiotics in husbandry and aquaculture leaded to the pollution of eco-environment. Thus, it is important to develop simple facile methods and effective functional materials for quick on-site analysis of antibiotics. Covalent organic frameworks (COFs), as a kind of porous crystalline covalent bond linked polymers, have demonstrated its power in multiple fields. Herein, we will discuss COFs-based materials utilized as antibiotics sensors with fluorescence method. For each sensor, we will mainly discuss the mechanism for antibiotics recognition, the preparation, characterization and fluorescence sensing performance of specific antibiotics. The mechanism to illustrate the interaction between sensors and antibiotics analytes would also be stressed.

## Graphical abstract

**Figure:**
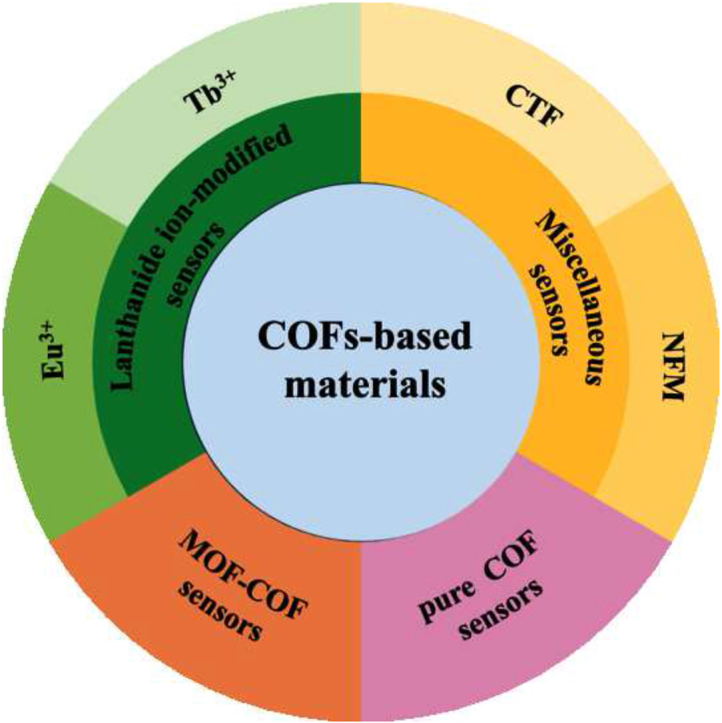

## 1 Introduction

**Scheme 1:**
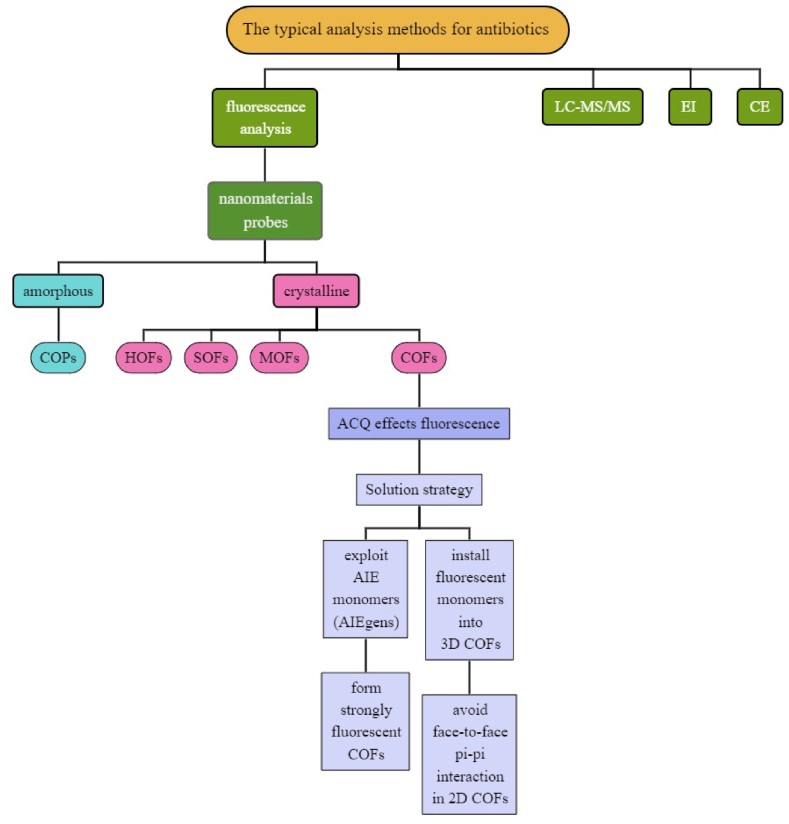
The important progress in the antibiotics detection of COFs.

Nowadays, there are two severe issues facing every country, which need to be addressed as soon as possible, energy crisis and environment pollution. As a kind of typical contaminants in water environment, antibiotics pose severe threat to the public safety [1]. Thus, rapidly on-site recognition and accurate quantification of antibiotics is an urgent demand [2]. The typical analysis methods for antibiotics mainly rely on liquid chromatography-tandem mass spectrometry (LC-MS/MS) [3], enzymatic immunoassay (EI) [4], capillary electrophoresis (CE) [5], and fluorescence analysis [6,7]. Compared with the other three methods, fluorescence-based methods do not depend on complex and expensive instrument, do not need large amount of eluent, and do not require rigorous pH, temperature and oxygen conditions [8]. Moreover, fluorescence-based method often has comparable level of sensitivity and lowest detection limit (LOD) with the former three methods, commonly in μM to nM concentration [9]. The high selectivity is another advantage. By elaborately designing the recognition groups, fluorophores and connecting groups, highly selective fluorescence probes, which respond only to a certain analyte, can be achieved [10]. Sometimes, even naked-eye detection can be realized by fluorescence analysis, which makes the on-site detection possible and largely reduces the instrumental operating costs [11]. Since most antibiotics do not possess relatively strong fluorescence emission in near-UV and visible-light region, its fluorescence detection relies on the fluorescence of sensors, which should exhibit considerable fluorescence intensity changes or emission wavelength shifts. The fluorescent detection of antibiotics usually uses organic small molecule dye as probe. Nanomaterials (nanoparticles/colloids or quantum dots, carbon dots), graphene, molecularly imprinted polymers can also be used as electrochemical sensors for the detection of various antibiotics. However, due to their high solubility in common solvents and relatively high toxicity, it is urgently desired to develop non-soluble and easy-to-reuse nanomaterials probes [12]. To meet this demand, multiple novel emerging nanomaterials were exploited. According to the crystallinity, these nano probes can be divided into two categories, amorphous and crystalline. Covalent organic polymers (COPs) is the typical representative of the former [13]. They can be facilely prepared by Suzuki-Miyaura, Sonogashira-Hagihara or Yamamoto coupling. And their fluorescence property can be fine-tuned by pre-installation or post-modification of the fluorophore groups. They often possess high surface area, large pore volume and rich specific recognition sites for sufficient binding interaction with antibiotics. The latter case include metal organic frameworks (MOFs) [14], covalent organic frameworks (COFs) [13], supramolecular organic frameworks (SOFs) [15] and hydrogen-bonding organic frameworks (HOFs) [16]. These framework materials commonly have long-range order, crystallinity, large surface area, high porosity [17]. They differ in the bonding manner in its reticular chemistry, MOFs are connected by coordination bonds, while COFs by covalent bonds, SOFs by supramolecular interaction and HOFs by hydrogen bonds. Among them, COFs are distinct from the other frameworks materials in that COFs’ connecting covalent bonds are much stronger than coordination, supramolecular interaction and hydrogen bonding [18]. Thus, COFs possesses highest stability among all the frameworks materials. Similar as amorphous COPs materials, COFs have considerable large surface area and pore volume. Moreover, COFs have comparable stability with amorphous polymers because of the strong connecting covalent bonds [19]. Besides, COFs share the advantages of frameworks materials, such as crystallinity, which is especially important for clarification of the relationship between structure and function [20]. COFs clear and confirmed structures provides great assistance in the prediction and design of achieving a specific function such as electrode material [21], catalysis [22], photovoltaics [23,24] and fluorescence analysis [25]. Usually, COFs do not dissolve in organic solvents or water, they can only disperse in some organic solvents. Thus, it is challenging to render it fluorescence because of the aggregation-caused-quenching (ACQ) effect [26,27]. To address this issue, multiple strategies are adopted. The first kind is to exploit aggregation-induced-emission (AIE) monomers (AIEgens) to form strongly fluorescent COFs [26]. The second is to install fluorescent monomers into three-dimensional COFs to avoid face-to-face pi-pi interaction in two-dimensional COFs which induces the ACQ effect [28]. There are also other types of fluorescence COFs. They have already exhibited utility in multiple fields in analytical chemistry, including the detection of explosives [25], metal ions [13], anions [13], biological important molecules [29], amines [30], volatile organic compounds [30] and antibiotics [31]. There have been other excellent reviews of fluorescent COFs as sensing probes [13,25], but there have been no articles focusing on antibiotics as analytes, so our content will specifically discuss the analysis of antibiotics by COFs probes. The progress is illuminated in Scheme 1.

Accurate and rapid detection of pesticide and veterinary drug residues, illegal additives, mycotoxins, antibiotics and other trace small molecules is an important content of food chemistry research. The development of rapid detection technology of trace small molecules is the development goal of food field detection. In recent years, emerging advanced materials with their unique physical and chemical properties have been gradually used for sample pretreatment and rapid detection of trace small molecules in food, with great application potential [32]. Antibiotics serve as main medicines to help people to counter the infection of numerous bacteria [33]. Since the discovery and separation of the first antibiotics in 1929, penicillin, which functions as an excellent drug to cure the gonorrhea and syphilis, which are incurable diseases before the prevalently spread of penicillin. After the World War II, numerous antibiotics were discovered and developed. However, due to the excessive use of antibiotics for medical, husbandry and aquaculture use, large amounts of untreated waste water with antibiotics residues are directly discharged into the water system in urban and rural area. These antibiotics residues threaten people's life because of its high toxicity, lipophilicity and bio-accumulation effect along the food chain [34]. Thus, it is important to develop effective sensing materials for its rapidly recognition and quantitatively analysis [35]. Statistics on antimicrobial resistance and antiviral infection show that the number of cases worldwide is increasing exponentially. In particular over the past few decades the need for traditional strategies such as developing alternatives rather than continuing to use antibiotics has not been met. Porous materials such as MOFs and COFs allow engineering coordination, heterogeneous catalysis, ion exchange, controllable targeted drug delivery, energetics, etc., due to their ideal properties, including excellent surface area, structural variability, and crystal structure richness. Shows the appropriate potential for specific virus detection. These frameworks can be used to package antibiotics or antivirals to fight pathogens; COFs have also been studied for photodynamic inactivation of pathogens [36].

The following discussion will be divided according to the types of the COFs-based materials structures including metal ion@COFs, MOF@COFs, pure COFs and miscellaneous probes. The final part is the summary, in which the main challenge and future developing direction in this research field will be concentrated.

## 2 COFs fluorescence sensing material for the detection of antibiotics

As above mentioned in the introduction part, COFs-based fluorescence materials can function as potent platforms for antibiotics sensing. Herein, we will discuss every sensor adopted for antibiotics detection in detail. According to the differences in sensing materials, we divide the subsequent discussion in four parts: lanthanide ion-modified COFs, MOF-COF complex, pure COFs and miscellaneous sensors.

In the interaction of fluorescent COFs sensors with target analytes, several types of sensing mechanisms have been developed, including photo-induced electron transfer (PET), fluorescence resonance energy transfer (FRET), intramolecular charge transfer (ICT), aggregation-induced emission (AIE), inner filter effect (IFE) and excited-state intramolecular proton transfer (ESIPT). PET refers to the phenomenon of intramolecular or intermolecular transfer of electrons induced by light. When the incident light illuminates the probe molecule, the donor molecule emits no or only weak fluorescence due to the transfer of electrons from the donor to the excited fluorophore. When the probe binds to the analyte, photoinduced electron transfer is blocked and the fluorophore resumes fluorescence emission. FRET means that when two fluorescence chromatophores are close enough, when the donor molecule absorbs a photon of a certain frequency, it is excited to a higher electron energy state, and before the electron returns to the ground state, the energy transfer is achieved by dipole interaction to the neighboring receptor molecule. ICT is based on the principle of intramolecular charge transfer, the core of which is the transfer of electrons from one part of the molecule to another. This charge transfer process causes the excited state of the molecule to change, which causes the change of fluorescence emission. The fluorescence probe is usually composed of two parts: the donor part has a high electron affinity, and the acceptor part has a high ionization energy. AIE refers to the phenomenon of enhanced luminescence of organic molecules after aggregation in high concentration solution, which is related to the concentration quenching effect and the fluorescence quenching effect caused by aggregation. IFE refers to the phenomenon that the fluorescence is weakened due to the absorption of excited or emitted light by the fluorescence or other absorbent substances when the concentration of the fluorescence is large or coexisting with other absorbent substances. ESIPT refers to the proton transfer reaction between the proton donor and the proton acceptor in the excited state when the probe molecule is excited by light.

In this paper, the mechanism of detecting antibiotics with COF material is introduced, and probes related to the mechanism are introduced in the following contents. Different sensing mechanisms of COFs for antibiotic detection are shown in Table 1.

**Table 1:** Different sensing mechanisms of COFs for antibiotic detection.

	Mechanisms
PET	intramolecular or intermolecular transfer of electrons induced by light
FRET	dipole interaction (no emission of light involved)
ICT	Intramolecular charge transfer causes changes in fluorescence emission
AIE	the phenomenon of enhanced luminescence of organic molecules after aggregation in high concentration solution
IFE	radiative re-absorption; light is first emitted and then re-absorbed by the second species
ESIPT	A proton transfer reaction between an excited proton donor and a proton acceptor

### 2.1 Lanthanide ion-modified COFs sensors

Some specific lanthanide ions can provide narrow emission spectrum with large Stokes shifts through energy-transfer induced D→F transition, from which generates long-time delayed fluorescence with τ in the range of several to hundred μs [37]. Mostly, Eu^3+^ and Tb^3+^ [38] are the most inclined ions to provide such sensitized fluorescence. However, commonly Eu^3+^ and Tb^3+^-based materials are usually unstable, easy to photo-bleach and possess narrow light-absorbing range and low photon efficiency. Covalent organic frameworks can function as stable matrix to anchor Eu^3+^ and Tb^3+^ by COFs’ imine or pyridine N group [39]. Because of the insolubility, hydrophobicity and multi-layer pi-pi stacking property of COFs, Eu^3+^ and Tb^3+^ anchoring in COFs is difficult to leach, and have high resistivity to photo-bleach.

**Fig. 1:**
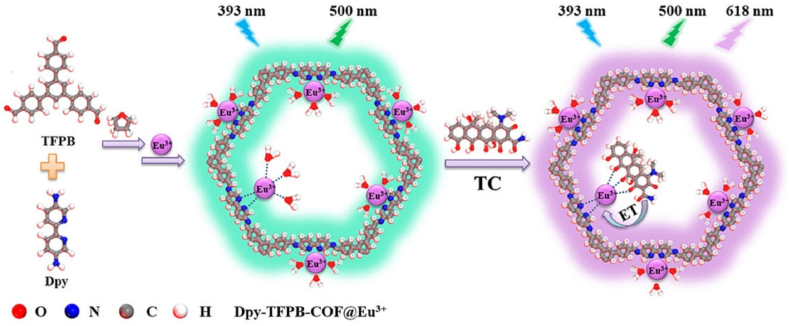
Schematic representation for the synthesis of Dpy-TFPB-COF@Eu^3+^ and its application selective and sensitive monitoring of TC (Copied with the permission from Elsevier 2023 [40].). (TFPB: 1,3,5-tris(*p*-formylphenyl)benzene, Dpy: 5,5′ -diamine-2,2′ -bipyridine, TC: tetracycline, ET: energy transfer.).

Yu et al. reported a Eu^3+^-modified bipyridine DPy-TFPB-COF [38], which acted as an efficient tetracycline ratiometric fluorescent sensor as illustrated in Fig. 1. Eu^3+^ was successfully branched inside COF's internal pore bipyridine N site by soaking COF in a Eu^3+^ solution. In the absence of tetracycline, the Eu^3+^-COF only exhibited emission in 500 nm (green light) under 393 nm excitation, which originated from Dpy-TFPB-COF's intrinsic emission, Eu^3+^ did not fluoresce since the surrounding water molecules leaded to Eu^3+^ non-radiation quenching process. However, in the presence of tetracycline, due to the stronger coordination of tetracycline β-diketone moiety to Eu^3+^, tetracycline displaced the surrounding water molecules and transferred energy to Eu^3+^ under 393 nm excitation, to initiate Eu^3+^ D→F fluorescence, which was called an antenna process. While Dpy-TFPB-COF still emitted the green light, which acted as a reference for this ratiometric fluorescence sensing. The lowest detection limit was as low as 7.5 nM. Moreover, this sensor had outstanding recoveries 97.8%–99.0 % in the real milk samples. Also, the sensor showed satisfactory selectivity towards a series of amino acids, metal ions and other antibiotics.

Bian et al. designed and synthesized a novel covalent organic skeleton (M-TT-COF) [41]. As an “turn on” fluorescence sensor, M-TT-COF can continuously and selectively identify and detect Eu^3+^ and tetracycline antibiotics, while other metal ions, anions and antibiotics have no significant effect on selective detection performance. The sensitivity of the sensor to Eu^3+^ is 3.94 μM and to tetracycline antibiotics (oxytetracycline) is 0.56 μM. In addition, M-TT-COF and Eu@M-TT-COF have good recoveries for Eu^3+^ and tetracyclines in actual water samples, respectively. The mechanism of fluorescence enhancement is attributed to the energy transfer from β-diketone structure to Eu^3+^. This work has successfully applied the covalent organic skeleton as an “on” fluorescence sensor for the identification and detection of rare earth metal ions and antibiotics.

**Fig. 2:**
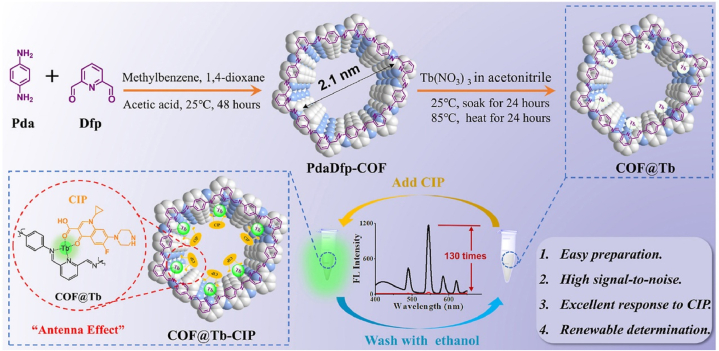
The schematic illustration of synthesizing route of COF@Tb and its renewable detection of CIP (Copied with the permission of Elsevier 2021 [42].). (Pda: P-phenylenediamine, Dfp: 2,6-Diformylpyridine.).

Apart from Eu^3+^ modified COFs, other fluorescent Ln^3+^ also synergistically function with COFs materials as antibiotics sensors. Tao et al. reported a COF@Tb as a turn-on fluorescent sensor for ciprofloxacin (CIP) as shown in Fig. 2 [42]. This sensing materials were prepared by post-modification. Initially, *p*-benzenediamine (Pda) and 2,6-diformylpyridine (Dfp) were condensed by a Schiff-base reaction to form Pda-Dfp-COF. Then, Tb^3+^ was chelated with COF's pyridine and imine N atom site by a soaking procedure. The as-formed COF@Tb exhibited no fluorescence under 330 nm excitation. However, when CIP was introduced into the solution, strong green fluorescence was observed even with naked eyes. The CIP acted as an antenna to sensitize Tb^3+^ D→F fluorescence similarly as the previous Eu^3+^@COF example. And this turn-on sensor has ultra-high sensitivity with a 3.01 nM LOD and excellent selectivity uninfluenced in the presence of a variety of other antibiotics including chloramphenicol, β-lactams, erythromycin, aminoglycoside and lincomycin. Moreover, this sensor had good reusability and could be reused after simply ethanol washing and did not show apparent leaching of Tb^3+^. Three types of real samples were tested with this sensor including milk, serum and tablets all providing good recoveries.

**Fig. 3:**
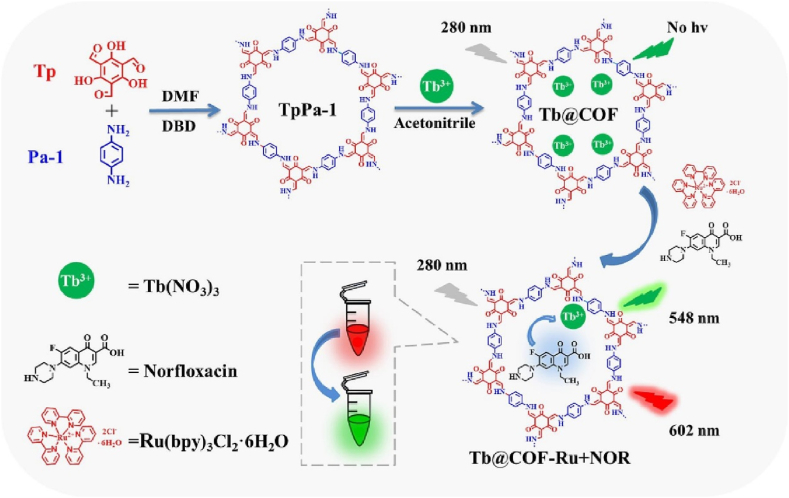
Schematic diagram of Tb@COF synthesis and visual detection of NOR (Copied with the permission of Elsevier 2023 [43].). (Tp: 2,4,6-Trihydroxybenzene-1,3,5-tricarbaldehyde, Pa-1: P-phenylenediamine.).

Besides ratiometric and turn-on fluorescence sensor, Hou et al. furthermore developed a multi-color fluorescence sensing platform for visual detection of norfloxacin (NOR) by a Tb@COF-Ru(bpy)_3_^2+^ as exhibited in Fig. 3 [43]. They prepared Tp-Pa-1-COF from 1,3,5-triformylphloroglucinol and *p*-benzenediamine. Tb was introduced by coordinating to COF's imine site. The exciting point of this work is that the authors introduced red-emitting Ru(bpy)_3_^2+^ besides fluorescence sensitive Tb. Upon excitation with ultraviolet light, Tb was sensitized by the chelating NOR and emitted green light. At the same time, Ru also emitted its own red light at 602 nm. The red and green color mixed into different color according to the NOR concentration. And this color was recorded by photographing and the image was further analyzed with a smartphone's color analyzer program to provide an RGB value, and according to the red to green ratio, the NOR concentration was read out from the corresponding working curve. In this way, an on-site rapid analysis of NOR in real samples was facilely realized only with a smartphone's App needless to use large instrument and complex data processor. Pharmaceuticals, water and different food samples were also analyzed with good spiked recoveries by this method.

### 2.2 MOF-COF sensors

Metal-organic-frameworks (MOFs) are a kind of inorganic-organic hybrid framework materials, which is formed by coordination bond between metal ions and organic ligands. Since it contained organic components, it was facile to tune the function of MOFs materials. Some MOFs materials exhibited strong fluorescence in visible region and can be used as luminescence sensors. Conjugating fluorescent MOFs with more porous and stable COFs materials can realize simultaneous sensing and removal of antibiotics.

**Fig. 4:**
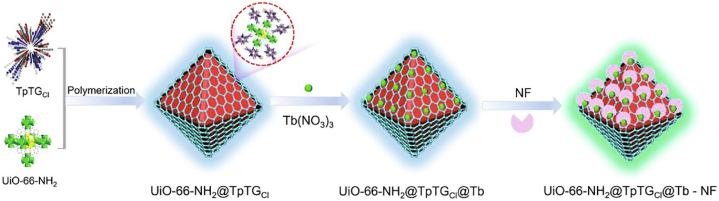
Schematic representation for the synthesis of UiO-66-NH_2_@TpTG_Cl_@Tb and its application for monitoring and capturing of nitrofurans (Copied with the permission from Elsevier 2022 [44].). (TpTG_Cl_: a guanidine-based ionic covalent organic framework, Uio-66-NH_2_: a Zr-based metal organic framework, NF: Norfloxacin).

Chen et al. reported a UiO-66-NH_2_@TpTG_Cl_@Tb^3+^ composite materials applied as efficient sensor and cleaner for norfloxacin in water environmental shown in Fig. 4 [44]. Initially, a guanidinium-based ionic COF was prepared in-situ in the presence of UiO-66-NH_2_ MOF material. And Tb^3+^ ion was further incorporated into the composite by hydrothermal method. The UiO-66-NH_2_@TpTG_Cl_ exhibited an emission peak at 450 nm, while Tb^3+^ emitted at 480 and 549 nm. With the addition of norfloxacin, the emission at 480 and 549 nm were intensified, while emission at 450 nm was not influenced. Thus, a ratiometric fluorescence probe system was established. The concentration of norfloxacin could be obtained from the linear equation of I_549_/I_450_. This composite sensor had high sensitivity, selectivity and low LOD. Furthermore, due to the presence of highly porous ionic COF component and the cationic property of ionic COF and Tb^3+^, this composite material was adopted to test the adsorptive removal property of norfloxacin, which was rich of anionic carboxyl group. Adsorption experiments indicated that the composite sensor possessed good adsorption ability of norfloxacin with high adsorptive capability and rapid adsorption equilibrium. The authors further explored the sensing mechanism using XPS experimental and DFT theoretical methods. XPS experiments exhibited strong bonding interaction between norfloxacin and Tb^3+^ in UiO-66-NH_2_@TpTG_Cl_@Tb^3+^. And DFT theoretical calculations displayed efficient electron transfer between norfloxacin and UiO-66-NH_2_@TpTG_Cl_@Tb^3+^ indicating strong interactions between sensor and analyte. This report was first to illustrate a MOF@COF composite fluorescence sensor for antibiotics detection.

**Fig. 5:**
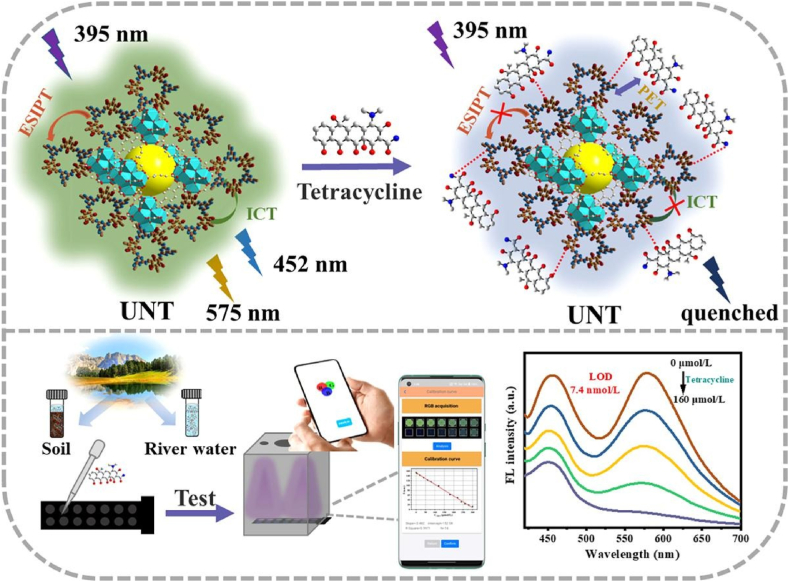
Schematic representations of UiO-66-NH_2_@TpTt-COF(UNT) core-shell composite for sensitive and optosmart sensing of tetracycline. (Copied with the permission from Elsevier 2023 [45].).

Different from the previous MOF@COF example, Li et al. reported a fluorescent TpTt-COF which was grafted by UiO-66-NH_2_(MOF) schematized in Fig. 5 [42]. Before the grafting procedure, TpTt-COF exhibited apparent fluorescence at 445 and 575 nm. And after incorporating UiO-66-NH_2_, both peaks were enhanced and the 445 nm emission peak red-shifted to 452 nm due to intermolecular hydrogen bond-accelerated intramolecular charge transfer (ICT) process between MOF's benzene and COF's triazine rings. The emission enhancement may possibly be ascribed to enhanced excited-state intramolecular proton transfer (ESIPT) and ICT. The as-synthesized UiO-66-NH_2_@TpTt-COF (UNT) was fully characterized with multiple techniques including SEM, TEM, PXRD, FT-IR, XPS, zeta-potential test to confirm its structure. UNT's dual emission property was utilized for the quantitative detection of tetracycline in water. Due to the photo-induced electron transfer (PET) effect between tetracycline and UNT, the emission was reduced upon tetracycline adding. The sensor exhibited high sensitivity, high selectivity, low LOD and high anti-interference. Furthermore, the authors explored the sensing mechanism. UV–Vis, FT-IR and XPS experimental methods were adopted. UV–Vis results indicated inner filter effect (IFE) but not Förster resonance energy transfer (FRET) contributed to fluorescence quenching. While FT-IR results displayed the strong intermolecular hydrogen bonding between tetracycline and UNT, demonstrating reduced ESIPT and ICT-induced fluorescence. XPS results further proved the intermolecular hydrogen bonding between tetracycline NH/OH group and UNT triazine C=N group. This interaction was the main cause for the fluorescence quenching. This sensor was also tested to be successfully adopted for actual sample analysis in spiked experiments with good recoveries. In all, Li's report was the first to exhibit a fluorescent COF can incorporate MOF for dual-emission and detection of an important antibiotics.

### 2.3 Pure COF sensors

Using only COFs as fluorescence sensors in the absence of other components like metal ions or MOFs is a challenging task due to the weak emission property of COFs. Due to the common eclipsed two-dimensional COF's π-π stacking structure, this intralayer non-bonding van der Waals interaction was easy to induce aggregation-caused-quenching (ACQ), rendering the COFs materials less emissive. Since the adoption of metal ions and MOFs may bring the corresponding secondary heavy-metal pollution due to metal leaching, it was desired to develop non-metal pure COFs fluorescence sensors for antibiotics detection in water environments.

**Fig. 6:**
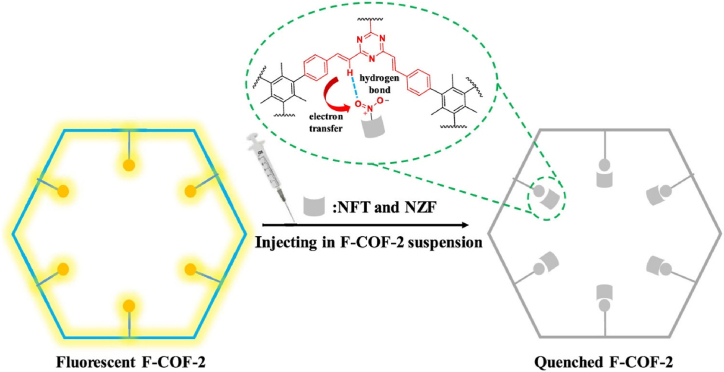
Mechanism diagram of antibiotic detection in F-COF-2. The formation of the ground-state nonfluorescent complex of F-COF-2 with nitrofurantoin and nitrofurazone is accompanied by electron transfer (Copied with the permission from ACS 2022 [46].). (NFT: nitrofurantoin, NZF: nitrofurazone.).

Huang et al. reported F-COF-2, which was synthesized with an AIEgen TST by a Knoevenagel reaction forming a two-dimensional conjugated COF [46]. This COF had stereoscopic methyl group in the center triazine ring, which rendered the COF both skeleton and spatial rigidification effect. The COF exhibited strong fluorescence and exhibited extremely high photoluminescence quantum yield, 23.4 % in solid state. Furthermore, this COF was utilized for the fluorescence detection of nitrofurantoin and nitrofurazone antibiotics. Due to its multiple binding site with antibiotics, the COF formed stable non-radiative ground-state complex with nitrofurans inducing efficient static quenching. Otherwise, IFE, PET and dynamic quenching also contributed to the effective fluorescence quenching and sensing of antibiotics. The mechanism was shown in Fig. 6. This report provided an effective strategy to construct highly emissive COFs by spatial rigidification for efficient antibiotics fluorescence sensing.

**Fig. 7:**
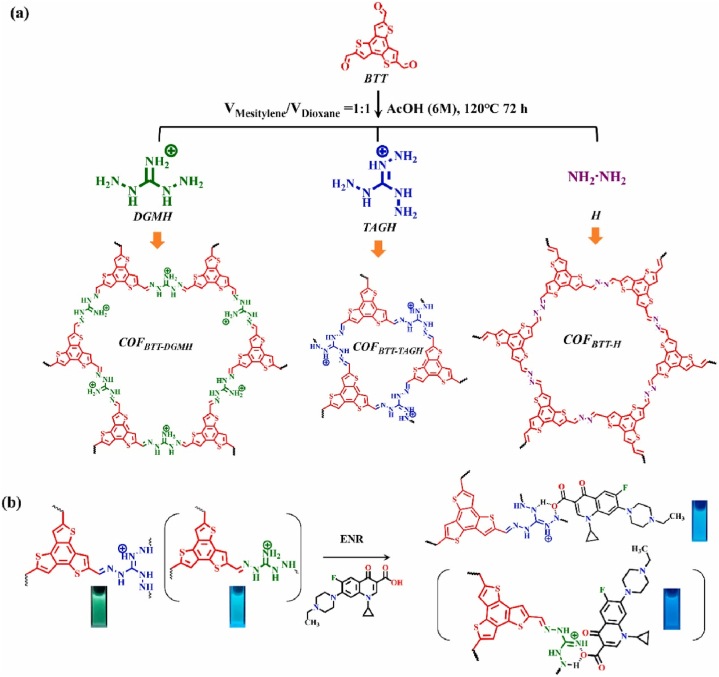
Schematic diagram of (a) Synthesis route of COF_BTT-DGMH_, COF_BTT-TAGH_ and COF_BTT-H_ as well as (b) the principle and process of ENR assay (Copied with the permission from Elsevier 2022 [47].). (BTT: benzotrithiophene tricarbaldehyde, DMGH: 1,3-diaminoguanidine monohydrochloride, TCGH: triaminoguanidine hydrochloride, ENR: Enrofloxacin.).

Song et al. reported COF_BTT-DGMH_ and COF_BTT-TAGH_ with cyan and green fluorescence [47]. Initially, they were synthesized by a Schiff-base reaction with benzotrithiophene (BTT) unit and guanidinium units (DGMH and TAGH) shown in Fig. 7. After careful characterization with multiple techniques including FT-IR, PXRD, N_2_ isosorption and SEM, their structures were thoroughly confirmed. Furthermore, their fluorescence property was explored. Different from COF_BTT-DGMH_ which was C_6_ symmetric possessing two emission peaks at 454 and 536 nm, C_3_ symmetric COF_BTT-TAGH_ only possessed only single emission peak at 540 nm. Upon enrofloxacin adding, the emission peak at 451 nm was extremely intensified, while the emission at 540 nm was basically uninfluenced. Thus, the enrofloxacin concentration was accurately quantified by a ratiometric fluorescence method from calculating I_454_/I_536_ and I_451_/I_540_. Since enrofloxacin exhibited weak fluorescence in solution but very strong emission in solid and viscous solution in 451 nm. This fluorescence “turn-on” might originate from the aggregation-induced-emission property of enrofloxacin. Furthermore, since the great fluorescence color contrast before and after enrofloxacin addition, COF_BTT-TAGH_ was prepared in the form as test paper and test gel adopted to visually detect enrofloxacin in the actual samples such as fish and clam metabolites. Just with a smart-phone App, the quantity of enrofloxacin residues in these samples were obtained and agreed with the value from HPLC method. Song's report adopted AIE mechanism and first realized fluorescence “turn-on” mode sensing for antibiotics by COFs materials.

Wang et al. developed two polyimide COF-1 and COF-2 by condensation of perylenebisimide anhydride with melem and melamine [48]. They adopted a green solvent-free method to synthesize these COFs only by grinding and calcination. The as-synthesized COFs were characterized by common techniques including PXRD, FT-IR, N_2_ isosorption, SEM, XPS and contact angle test to provide evidences for the successful construction of these COFs as proposed. Furthermore, their fluorescence properties were explored in different solutions. DMF and alkaline aqueous solution environment were found to be optimal for fluorescence emission. Then, COF-1 and COF-2 were applied for the detection of metal-ion in water and antibiotics in DMF. The experimental results showed that multiple transition-metal cations effectively quenched the COFs fluorescence since the metal ions interacted with hydroxide anions and formed metal-hydroxide complexes. Thus, the COFs fluorescence would be greatly reduced due to aggregation-caused-quenching in less alkaline solutions. The optimal pH for COFs fluorescence was found to be 10 to inhibit ACQ effect. And ofloxacin and tetracycline antibiotics dissolved in DMF were also found to be efficient fluorescence quencher for COF-1 because these antibiotics were rich of benzene rings. The π-π interactions between COF-1 and these antibiotics contributed to the quenching of COF fluorescence. The COF-1 possessed high sensitivity, low LOD and wide-spectrum compatibility for the detection of transition-metal cations. In all, this report was first to prove that pure COFs fluorescence sensors possessed the ability for either metal ions and antibiotics analysis.

Wang et al., for the first time, synthesized a high-crystallinity fluorescent covalent organic skeleton functionalized with phenylboric acid (BA@COF), which can be used for rifamycin antibiotics and water sensing and rifamycin antibiotic adsorption [49]. In order to effectively form BA@COF by synthetic modification, fluorescent COF(COF-OME) was prepared with deep eutectic solvent without using catalyst. BA@COF has good fluorescence properties and adsorption capacity, and has good detection and adsorption effect on rifampicin, rifapentine and rifambutin. The study of fluorescence quenching mechanism shows that internal filtration effect, dynamic quenching and photoinduced electron transfer are the main quenching mechanisms. In addition, BA@COF showed good selectivity for three antibiotics, mainly due to the pore selectivity of BA@COF, hydrogen bonding and π-π interactions. In summary, a dual-function platform based on BA@COF was successfully developed for the detection and adsorption of three antibiotics in different samples. In addition, BA@COF has a good effect on the detection of moisture in ethanol.

Lin et al. proposed a solvent-free and simple mechanochemical method for the synthesis of COFs. The prepared sulfonate-containing COF TpBD-(SO_3_Na)_2_ has good thermal and chemical stability, and can be used as an effective adsorbent for selective trapping fluoroquinolone antibiotics (FQs) with its ionic interface and abundant negative charge sites [50]. The maximum adsorption capacities of norfloxacin, enoxacin and ciprofloxacin were 1709.6, 1661.5 and 1362.8 mgg−1, respectively. The adsorption mechanism of TpBD-(SO_3_Na)_2_ and FQs was further investigated through characterization and density functional theory calculations. The synergistic adsorption of TPBD-(SO_3_NA)_2_ and FQS was proved, including π-π* interaction, electrostatic interaction and hydrogen bonding. In addition, based on the fluorescence signal of sodium disulfonate TpBD-(SO_3_Na)_2_, the FQs fluorescence detection platform was established. The detection limit of norfloxacin was 8 μmol/L, which was linear in the range of 0.03∼6 μmol/L. In conclusion, this study provides a new strategy for the green synthesis of COFs and expands its application in the simultaneous detection and removal of FQs in aquatic environments.

### 2.4 Miscellaneous

Apart from metal-ion@COF, MOF@COF and pure COFs fluorescence sensors, there also appeared some other related sensors for antibiotics detection such as covalent triazine framework (CTF)-based and nanofiber mat (NFM)-COF-based probes.

**Fig. 8:**
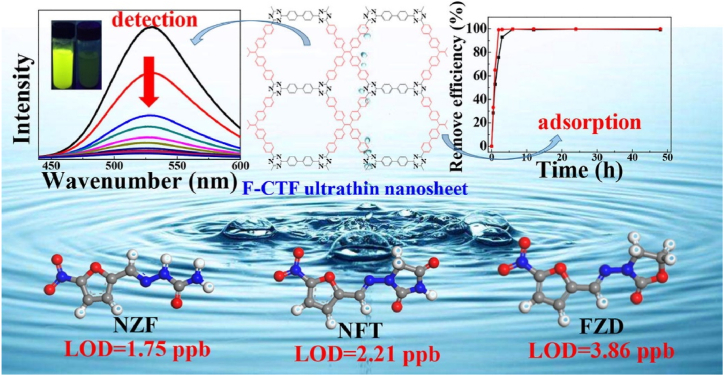
Schematic diagram of the simultaneous fluorescence detection and adsorption of nitrofurans antibiotics by covalent triazine framework materials (Copied with the permission of Elsevier 2019 [51].). (NZF: nitrofurazone, NFT: nitrofurantoin, FZD: furazolidone.).

Zhong et al. explored the fluorescence CTF polymer sensors for the detection of nitrofurans antibiotics as displayed in Fig. 8 [51]. Initially, a tetraphenylethylene-like ETTC monomer was condensed with phenamidine and diphenamidine to form two hexagonal CTFs materials. Due to the existence of tetraphenylethylene, ETTC and the two CTFs were rendered with AIEgen property. All of them exhibited bright green emission under UV light excitation. Because of the skeleton rigidification effect, CTFs possessed higher PLQY and larger k_rad._ than ETTC monomer. Both CTFs materials were thoroughly characterized with common techniques including PXRD, FT-IR, XPS spectra, ^13^C CP-MAS NMR, N_2_ isosorption. Furthermore, these two CTFs were utilized for further applications of nitrofurans antibiotics fluorescence sensing. Both of the F-CTFs exhibited high quenching efficiency even at very low antibiotics concentration in aqueous solution. Moreover, the sensors had large K_sv_, excellent selectivity and anti-interference property. Since these sensors had large surface area and pore volume, they were adopted to adsorb the nitrofurans antibiotics. F-CTFs exhibited high adsorption capacity and rapid adsorption equilibrium for nitrofurans antibiotics. The mechanistic experimental results indicated that the CTFs pores fabricated pre-concentration effect for ultra-low concentration fluorescence sensing of antibiotics. This report was the first to utilize CTFs materials for fluorescence detection of antibiotics. Due to the extreme stability of CTFs than COFs and MOFs, this report may provide useful and novel strategy to construct practical porous frameworks-based fluorescence sensors for antibiotics detection.

**Fig. 9:**
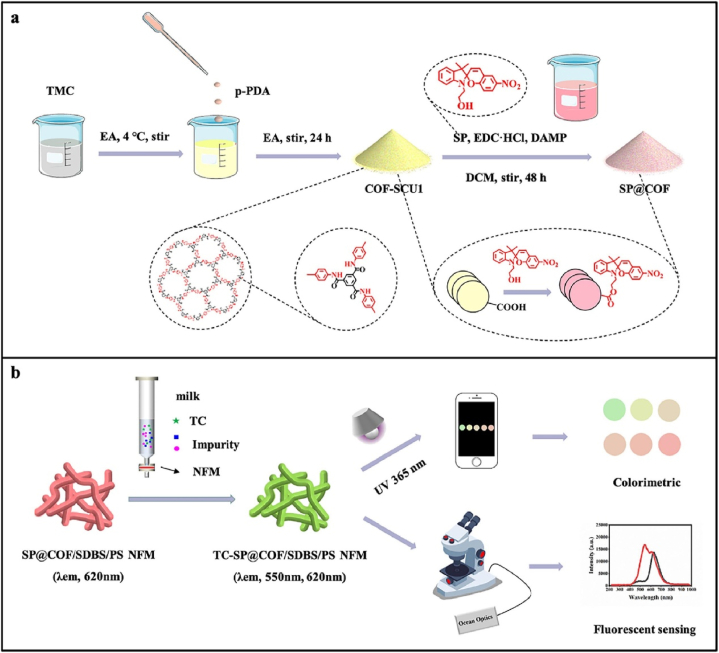
Schematic of (a) SP@COF synthesis and (b) Process of dynamic solid-phase sensing detection of TC by SP@COF/SDBS/PS NFM (Copied with the permission of Elsevier 2022 [52].). (TMC: Trimesoyl chloride, *p*-PDA: *p*-phenylenediamine, SP: 1-(2-hydroxyethyl)-3,3-dimethylindoline-6′-nitrobenzospiropyran, EDC^.^HCl: 1-ethyl-(3-dimethylaminopropyl) carbodiimide hydrochloride, DAMP: 4-dimethylaminopyridine, PS: polystyrene, SDBS: Sodium dodecyl benzene sulfonate, EA: Ethyl acetate, DCM: dichloromethane, TC: tetracycline, NFM: nanofiber mat.).

Xu et al. developed a nanofiber-mat SP@COF/SDBS/PS sensing materials for tetracycline ratiometric fluorescence detection as exhibited in Fig. 9 [47]. They used COF to improve the composite material's solvent stability and thermal stability. Initially, they prepared a spiropyran-modified amide-COF SP@COF-SCU-1 by solution-based post-modification of COF. The spiropyran component exhibited unique red emission at 620 nm of spiropyran fluorophore. Furthermore, the as-synthesized SP@COF was modified by an electro-spinning method. The SP@COF was added with PS (polystyrene) and SDBS (sodium dodecyl benzene sulfonate) stirred in DMF (dimethylformamide). And the mixed solution was proceeded with a special electro-spinning instrument with high electro-voltage pole and needle squeezing procedures. The as-prepared SP@COF/SDBS/PS was further characterized by SEM, XPS, FT-IR, contact angle tests and clearly confirmed its proposed structure. This composite material exhibited good adsorption efficiency for tetracycline. Moreover, the composite material could be used as ratiometric fluorescence sensor for tetracycline detection, because tetracycline induced a strong “turn-on” fluorescence at 550 nm, and spiropyran's 620 nm fluorescence was uninfluenced. Thus, the spiropyran's red emission was adopted as a reference for tetracycline detection. And this sensor could even realize naked-eye visual detection. The color changes from red to green after the addition of tetracycline was also observed by confocal laser scanning microscope (CLSM). The color change was read by a smartphone App to analyze R/G ratio obtaining a working curve to calculate tetracycline concentration. This sensor exhibited good selectivity and recoveries with spiked matrix samples. This report first indicated that COFs materials could be incorporated with fluorescent nanofiber-based materials and load unstable dyes for practical antibiotics detection.

Hu et al. developed an enzyme-regulated fluorescence sensor using a Fe_3_O_4_@COF/Fe^3+^ probe for chloramphenicol (CAP) determination [53]. The Fe_3_O_4_@COF synthesized by hydrothermal method has dual functions of magnetic carrier and signal probe. Bovine serum albumin is coupled with chloramphenicol, adsorbed on the surface of Fe_3_O_4_@COF, and binds to the CAP competing antibody. Antibodies interact with alkaline phosphatase through the biotin-streptavidin system. At the same time, ascorbic acid produced by enzyme catalyzed reaction led by alkaline phosphatase can effectively restore the fluorescence of Fe_3_O_4_@COF quenched by Fe^3+^. After experimental verification and gradual optimization, the log-linear relationship between CAP concentration and fluorescence intensity was established in the range of 2 × 10^−4^∼10 μgmL^−1^, and the detection limit was good (9.2 × 10^−5^ μgmL^−1^).In summary, this method provides a sensitive and reliable method for the accurate detection of CAP, and the results are in good agreement with HPLC and laboratory and natural CAP analysis results.

## 3 Summary

In this paper, the research progress of COF-based materials in the rapid detection of trace antibiotics in food safety and environmental governance was reviewed. The available data are sifted, research methods and results are studied and compared, and the most advanced and useful methods are introduced. The sensing methods and the latest development of these technologies are briefly introduced. The discussion mainly introduced different type of COFs-based sensing materials performances for this application. Table 2 provides an overview of COF sensors for antibiotic detection.

**Table 2:** COF-based sensors for antibiotic detection.

Sensors	Samples	Analytes	Recovery rate	Techniques	LOD^a^	Ref.
DPy-TFPB-COF@Eu^3+^	Water	TC^g^	97.8–99.0 %	b	7.5 nM	[40]
Eu@M-TT-COF	Water	TC^g^	87.90–100.12 %	b	560 nM	[41]
PdaDfp-COF@Tb	Milk, Serum, Tablets	CIP^h^	99.08–102.20 %	b	3.01 nM	[42]
Tb@COF-Ru(bpy)_3_^2+^	Pharmaceuticals	NOR^i^	90.8–109 %	b, c	7.3 nM, 330 nM	[43]
	Honey, Milk, Egg, Beef		88.4–107 %			
	Water		90.4–110 %			
UiO-66-NH_2_@TpTG_Cl_@ Tb^3+^	Soil	TC^g^	92.96–98.44 %	b	7.4 nM	[44]
	River water		88.58–104.31 %			
UiO-66-NH_2_@TpTt-COF@Tb	Water	NOR^i^	95–108 %	b	4 nM	[45]
F-COF-2	–	NFT^j^	–	b	3.35 ppb	[46]
		NZF^k^		c	9.64 ppb	
COF_BTT-DGMH_	–	ENR^l^	–	b	12.60 nM	[47]
COF_BTT-TAGH_	Fish		97.0–102.5	b	0.82 nM,	
				c	37.84 nM^d^	
	Clam		90.5–96.6	c	106.2 nM^e^	
				c	26 nM^f^	
COF-1	DMF	TC^g^	98 %	b	2000 nM	[48]
		OF^m^	110 %	b	6500 nM	
Ba@COF	Water	RIF^o^	75.20–123.46 %	b	0.03 μg/mL	[49]
		RFT^p^				
		RBT^q^				
TpBD-(SO_3_Na)_2_	Water	NOR^i^	86.0–114.0 %	b	8 nM	[50]
F-CTF-1	Water	NZF^k^	–	b	4.97 ppb	[51]
		NFT^j^	–	b	8.08 ppb	
		FZD^n^	–	b	13.35 ppb	
F-CTF-2	Water	NZF^k^		b	1.75 ppb	[51]
		NFT^j^		b	2.21 ppb	
		FZD^n^		b	3.86 ppb	
SP@COF/SDBS/PS NFM	Milk	TC^g^	94.66–103.66 %	b	2.14 nM	[52]
Fe_3_O_4_@COF/Fe^3+^	Milk, Water, Soil	CAP^s^	96.4–103.7 %	b	9.2 × 10^−5^ μg/mL	[53]

a: limit of detection. b: fluorescent probe. c: the combination of probe and smartphone. d: solution. e: test paper. f: test gel. g: tetracycline. h: ciprofloxacin. i: norfloxacin. j: nitrofurantoin. k: nitrofurazone. l: enrofloxacin. m: ofloxacin. n: furazolidone. o: rifampicin. p: rifapentine. q: rifabutin. r: enoxacin. s: chloramphenicol.

Apart from pure-COFs sensing materials, lanthanide metal ion@COFs, metal-organic-framework@COFs and other miscellaneous COFs-related fluorescence materials for antibiotics sensing were detailly described. The preparation, characterization, sensing performance and especially the mechanism of these COFs utilized for antibiotics fluorescence sensing were focused. Since Yaghi's first report of COF-1 and COF-5 in 2005, the development of COFs materials has already come a long way [54]. But its application in antibiotics sensing by fluorescence method is still in very infantile stage. The report of this field only emerged in recent several years. Antibiotics detection possessed the undoubtful importance in multiple field including environmental inspection, food safety and basic research. And fluorescence is one of the most promising method due to its many advantages including low cost, inexpensive instrument and facile testing procedures.

## 4 Outlook

COFs-based materials are also one of the most promising and novel fluorescence materials due to its tunability, porosity, stability, clear crystalline structure and excellent fluorescence performance. Although there are already some successful reports on this point, there are still several directions to be improved and some drawbacks should be overcome for better practical application. Initially, more turn-on fluorescence COFs-based sensors should be discovered and adopted for the antibiotics sensing application. It is apparent that recognizing a bright signal originating from a weak signal is much easier to observe a weak signal from the disappearance of a bright signal. If this goal could be realized, the naked-eye visual analysis of antibiotics in the field environment would become a routine process prohibiting present cumbersome sample preparation, expensive in-door instruments and high operating costs. Secondly, the recyclability and reusability of these COFs-based materials should be improved. Commonly, the COFs-based materials were discarded after single use in present laboratories. Since some COFs-based materials contains lanthanide-metal element like Tb and Eu, these toxic rare-earth cations would generate secondary heavy-metal pollution for aquaculture and drinking-water source. Besides, because COFs are commonly very valuable materials synthesized from expensive monomers, the discarding of them after single use is wasteful. Thus, it is highly desired to make these sensing materials into a sensing device. The setting up of a practical device would solve this issue from the root. Until now, there are still no reports on devitrification of COFs-based materials for antibiotics sensing application. Thirdly, it is highly desired to develop more COFs-based fluorescence probes in water environment. Nowadays, most COFs-based fluorescence sensors work in organic solvent and detect analytes like antibiotics in organic solvents such as DMF, methanol, ethanol or ethyl acetate. COFs exhibited much better dispersibility and compatibility in polar organic solvent than water. In aqueous system, the two-dimensional COFs inclined to adopt more stronger and condenser π-π stacking manner. In polar organic solvents, since COFs benzene rings have relatively stronger interaction with solvent molecule, the intralayer interaction in COFs itself is weaker. The more dispersed COFs in organic solvents exhibited much brighter fluorescence compared with the less dispersed COFs in water environment. This phenomenon was mainly due to the less aggregation-caused-quenching effect (ACQ) in organic solvents than water. Although COFs possessed better fluorescence and sensing performances in organic solvents, water is the ideal sensing medium for multiple valuable analytes including antibiotics. First, most real field environment analysis needs to be conducted in water environment. And water is the greenest solvent in comparison with all other organic solvents. Though the sensing of antibiotics by COFs-based fluorescence materials is just in budding-phase, the future of this field is bright due to its importance and value for porous materials and analytical sciences researches.

## Data availability statement

Not concerned data availability since it is a review.

## CRediT authorship contribution statement

**Mingyang Ji:** Writing – original draft. **Jiani Li:** Writing – original draft. **Anan Liu:** Writing – review & editing. **Dongge Ma:** Writing – review & editing, Writing – original draft.

## Declaration of competing interest

The authors declare no competing interests.
